# Involvement of the MEK-ERK/p38-CREB/c-fos signaling pathway in Kir channel inhibition-induced rat retinal Müller cell gliosis

**DOI:** 10.1038/s41598-017-01557-y

**Published:** 2017-05-03

**Authors:** Feng Gao, Fang Li, Yanying Miao, Lin-Jie Xu, Yuan Zhao, Qian Li, Sheng-Hai Zhang, Jihong Wu, Xing-Huai Sun, Zhongfeng Wang

**Affiliations:** 1 Department of Ophthalmology at Eye & ENT Hospital, Shanghai Key Laboratory of Visual Impairment and Restoration, Fudan University, Shanghai, 200031 China; 2 Institutes of Brain Science and State Key Laboratory of Medical Neurobiology, Collaborative Innovation Center for Brain Science, Fudan University, Shanghai, 200032 China

## Abstract

Our previous studies have demonstrated that activation of group I metabotropic glutamate receptors downregulated Kir channels in chronic ocular hypertension (COH) rats, thus contributing to Müller cell gliosis, characterized by upregulated expression of glial fibrillary acidic protein (GFAP). In the present study, we explored possible signaling pathways linking Kir channel inhibition and GFAP upregulation. In normal retinas, intravitreal injection of BaCl_2_ significantly increased GFAP expression in Müller cells, which was eliminated by co-injecting mitogen-activated protein kinase (MAPK) inhibitor U0126. The protein levels of phosphorylated extracellular signal-regulated protein kinase1/2 (p-ERK1/2) and its upstream regulator, p-MEK, were significantly increased, while the levels of phosphorylated c-Jun N-terminal kinase (p-JNK) and p38 kinase (p-p38) remained unchanged. Furthermore, the protein levels of phosphorylated cAMP response element binding protein (p-CREB) and c-fos were also increased, which were blocked by co-injecting ERK inhibitor FR180204. In purified cultured rat Müller cells, BaCl_2_ treatment induced similar changes in these protein levels apart from p-p38 levels and the p-p38:p38 ratio showing significant upregulation. Moreover, intravitreal injection of U0126 eliminated the upregulated GFAP expression in COH retinas. Together, these results suggest that Kir channel inhibition-induced Müller cell gliosis is mediated by the MEK-ERK/p38-CREB/c-fos signaling pathway.

## Introduction

Glaucoma, a blinding retinal disease, is characterized by vision loss resulting from apoptotic death of retinal ganglion cells (RGCs)^1–3^, and is regarded as a retinal neurodegenerative disease^4^. Included in the complicated pathogenesis of glaucoma, activated glial cells have been demonstrated to be involved in retinal neurodegeneration^5–11^. As a major type of glial cell in the vertebrate retina, Müller cells also undergo reactivation (gliosis) in a variety of retinal pathological disorders including glaucoma^5, 6, 11–17^. Activated Müller cells are characterized by upregulated expression of glial cytoskeletal proteins, such as glial fibrillary acidic protein (GFAP) and vimentin^11, 18–21^.

Previous reports have shown that inward rectifying K^+^ (Kir) currents were downregulated in retinal glial cells obtained from patients with glaucoma^18^. Our previous studies demonstrated that Kir currents, especially Kir4.1-mediated ones, and Kir4.1 proteins in Müller cells showed a significant reduction in a rat chronic ocular hypertension (COH) model due to over-activated group I metabotropic glutamate receptors (mGluR I) by excessive extracellular glutamate, which contributes to Müller cell gliosis^11^. In purified cultured Müller cells, we further demonstrated that dihydroxyphenylglycine (DHPG), an mGluR I agonist, may decrease functional Kir4.1 channels in the cell membrane by inhibiting Kir4.1 protein and mRNA levels, and subsequently inducing an increase in GFAP expression^22^. Although Müller cells express various subtypes of Kir channels, including Kir2.1, Kir4.1, and Kir 5.1^23–26^, Kir4.1, which is involved in Müller cell gliosis, may be selectively modulated. Since Kir channels with high K^+^ permeability are essential for maintaining a strongly hyperpolarized resting membrane potential for Müller cells to exert their physiological functions, inhibition of Kir channels leads to depolarization of the cell membrane and could result in a loss of its neuron-supportive functions^6, 23, 27, 28^.

Previous studies have shown that some intracellular signaling pathways may be activated in Müller cells under retinal pathological conditions. For instance, expression of phosphorylated extracellular signal-regulated protein kinase (p-ERK) in Müller cells was increased in a rat model of retinal ischemia-reperfusion^29^. In glaucomatous human eyes, both the immunostaining intensity of mitogen-activated protein kinases (MAPKs) and the number of MAPK-positive cells were greater than that in control eyes, and elevated expression of p-ERK was localized to glial cells^30^. However, it is not yet absolutely definite whether or which of these signal molecules are involved in Müller cell gliosis following glaucoma onset. In this study, we explored the underlying mechanisms that link Kir channel inhibition and upregulation of GFAP expression in rat retinal Müller cells.

## Results

### Involvement of the MAPK/ERK signaling pathway in Müller cell gliosis due to inhibition of Kir channels

**Figure 1:**
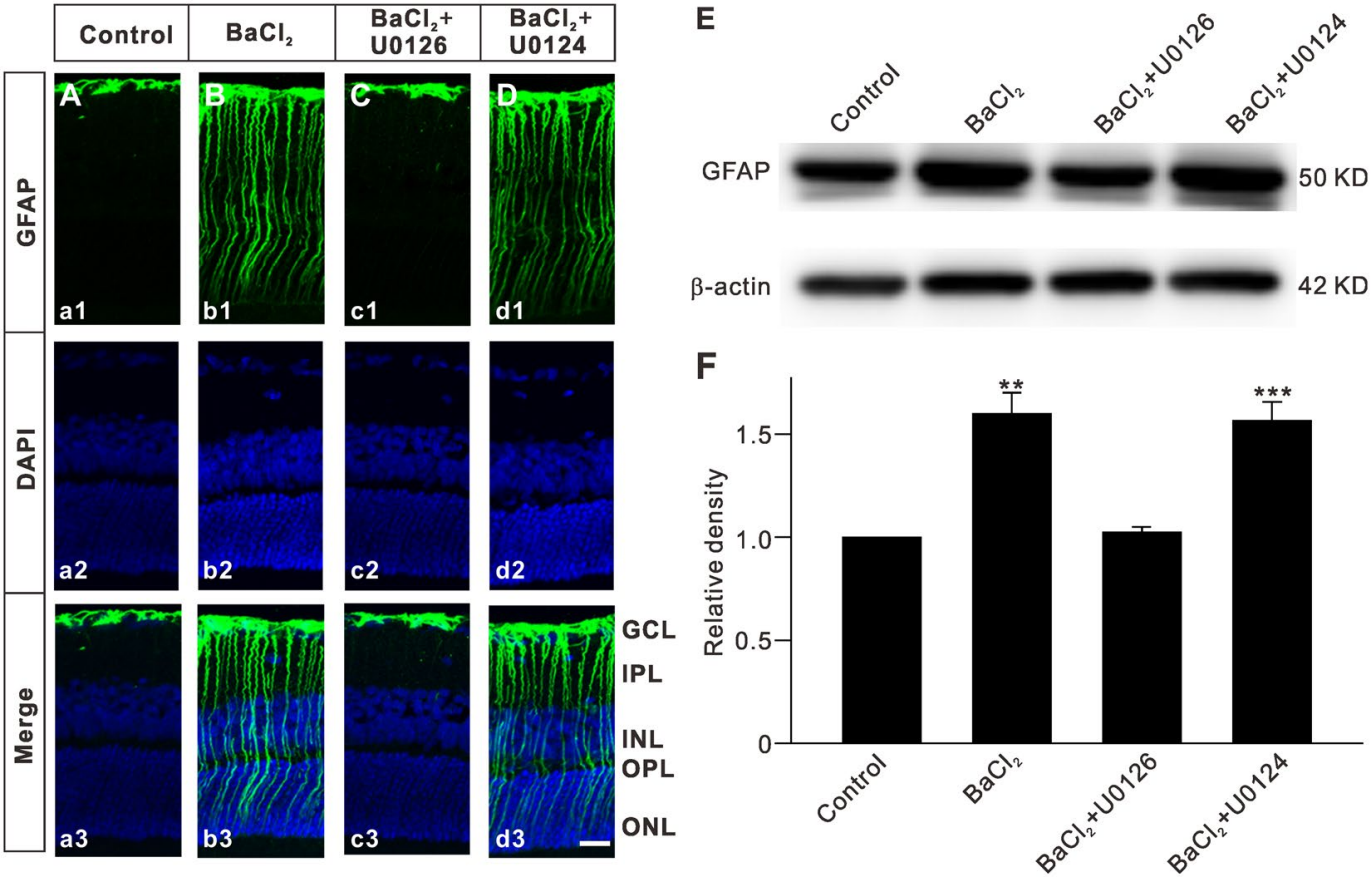
Inhibition of Kir channels induces an elevation of GFAP expression in Müller cells by activating MAPKs. Vehicle (0.9% saline), BaCl_2_, BaCl_2_ + U0126 or BaCl_2_ + U0124 was injected into the vitreous space in a volume of 2 μL, respectively. (**A**–**D**) Immunofluorescence labeling showing the changes in GFAP protein expression in rat retinal vertical slices taken from vehicle (Control) (Aa1), BaCl_2_ (Bb1), BaCl_2_ + U0126 (Cc1) or BaCl_2_ + U0124 (Dd1) injected retinas, respectively. a2–d2 are corresponding DAPI images and a3–d3 show the merged images. Scale bar, (for all the images) 20 µm. GCL, ganglion cell layer; IPL, Inner plexiform layer; INL, inner nuclear layer; OPL, outer plexiform layer; ONL, outer nuclear layer. (**E**) Representative immunoblots showing the changes in GFAP protein levels under different conditions as shown in (**A–D**). (**F**) Bar charts summarizing the average densitometric quantification of immunoreactive bands of GFAP protein expression under different conditions. All data are normalized to control. *n* = 6 for each group. **P < 0.01 and ***P < 0.001 *vs*. Control.

We first confirmed that inhibition of Kir channels by BaCl_2_ indeed induced upregulation of GFAP expression in normal retinas^11^. BaCl_2_ was injected intravitreally and retinas were collected 7 d after the injection for immunohistochemistry and Western blot analysis. As shown in Fig. 1A, GFAP expression was strictly localized to the endfeet of Müller cells in the ganglion cell layer (GCL) of the retinal section obtained from saline-injected eye (control) (a1 and a3). A significant increase in GFAP expression was observed in the section obtained from BaCl_2_-injected retina (Fig. 1B,b1 and b3). We then examined the possible involvement of MAPK signaling in BaCl_2_-induced upregulation of GFAP expression. The upregulation of GFAP expression was significantly reduced by co-injecting U0126, a MAPK inhibitor (Fig. 1C). Co-injection of U0124, an inactive analog of U0126, did not affect the BaCl_2_ effect on expression of GFAP (Fig. 1D). Consistently, Western blotting revealed that total GFAP protein levels extracted from BaCl_2_-injected retinas were profoundly increased, with an average protein density of 159.9 ± 10.3% that of controls (n = 6, *P* = 0.001), which was rescued by U0126 (102.5 ± 2.5% of control, n = 6, *P* = 0.647), but not U0124 (156.7 ± 9.0% of control, n = 6, *P* < 0.001 *vs*. control and *P* = 0.849 *vs*. BaCl_2_-injected retinas) (Fig. 1E,F). These results suggest that the MAPK signaling pathway is involved in Kir channel inhibition-induced upregulation of GFAP expression in Müller cells.

**Figure 2:**
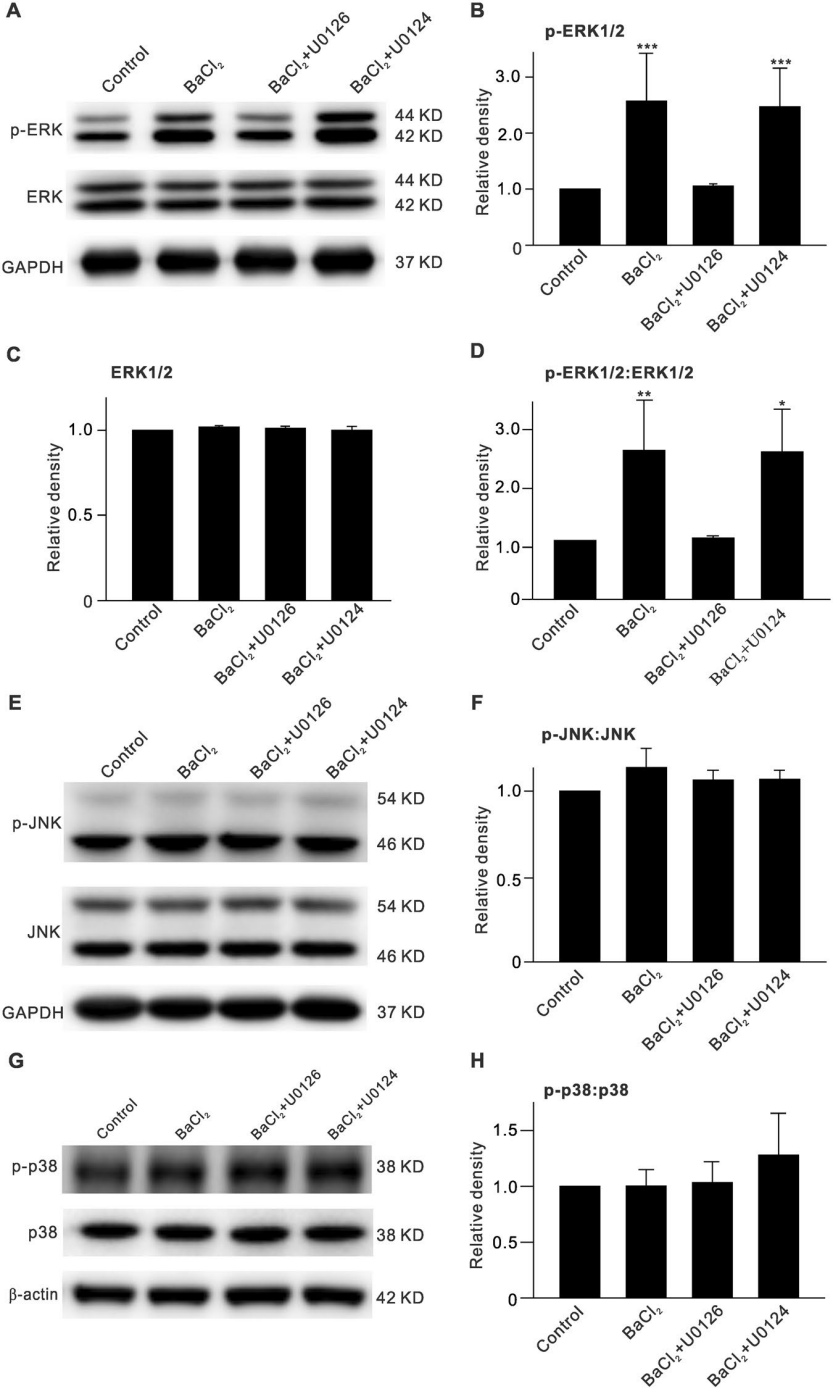
Increase of p-ERK expression in BaCl_2_-injected retinas. (**A**) Representative immunoblots showing changes in p-ERK1/2 and ERK1/2 levels in vehicle (Control)-, BaCl_2_-, BaCl_2_ + U0126- or BaCl_2_+U0124-injected retinas, respectively. (**B**,**C**) Bar charts summarizing the average densitometric quantification of immunoreactive bands of p-ERK1/2 (**B**) and ERK1/2 (**C**) protein expression under different conditions. (**D**) Bar charts summarizing the average p-ERK1/2:ERK1/2 ratios under different conditions. *n* = 6 all each group. *P < 0.05 and **P < 0.01 *vs*. Control. (**E**,**G**) Representative immunoblots showing changes in p-JNK and JNK levels (**E**), p-p38 and p38 (**G**) levels in vehicle (Control)-, BaCl_2_-, BaCl_2_ + U0126- or BaCl_2_ + U0124-injected retinas, respectively. (**F**,**H**) Bar charts summarizing the average p-JNK:JNK and p-p38:p38 ratios under different conditions. Note that inhibition of Kir channels by injecting BaCl_2_ did not affect p-JNK and p-p38 levels. *n* = 6 all each group.

The MAPKs include three members: ERK, c-Jun N-terminal kinase (JNK) and p38 kinase (p38)^31^. We tested which of these mediate the BaCl_2_-induced upregulation of GFAP expression. As shown in Fig. 2A, although total ERK1/2 level showed no significant changes after BaCl_2_ injection, p-ERK1/2 level was increased, as compared to control. The average ERK1/2 and p-ERK1/2 levels were 101.9 ± 0.1% (n = 6, *P* = 0.889) (Fig. 2C) and 256.8 ± 85.5% of control (n = 6, *P* < 0.001) (Fig. 2B), respectively, with the average p-ERK1/2:ERK1/2 ratio increased to 253.2 ± 85.8% of control (n = 6, *P* = 0.008) (Fig. 2D). The BaCl_2_-induced enhancement of p-ERK1/2 and p-ERK1/2:ERK1/2 ratio was rescued by intravitreal co-injection of U0126 to 105.5 ± 3.9% (n = 6, *P* = 0.828) and 104.3 ± 3.9% of control (n = 6, *P* = 0.874), respectively. However, co-injection of U0124 did not affect the BaCl_2_-induced effects on p-ERK1/2 levels (247.0 ± 69.0% of control, n = 6, *P* < 0.001) and p-ERK1/2:ERK1/2 ratio (250.6 ± 72.8% of control, n = 6, *P* = 0.011 *vs*. control and *P* = 0.813 *vs*. BaCl_2_ alone) (Fig. 2B,D). In contrast, JNK and p38 protein expression differed under these conditions. Compared with controls, no significant changes were observed in the average protein levels of JNK, p-JNK, p38, and p-p38, or in the average ratios of p-JNK:JNK and p-p38:p38 (n = 6 for each group, *P* all >0.05) in BaCl_2_-injected retinas, with or without co-injection of U0126 or U0124 (Fig. 2E–H). These results suggest that ERK, but not JNK or p38, was involved in the upregulation of GFAP expression in Müller cells when Kir channels were inhibited by BaCl_2_.

**Figure 3:**
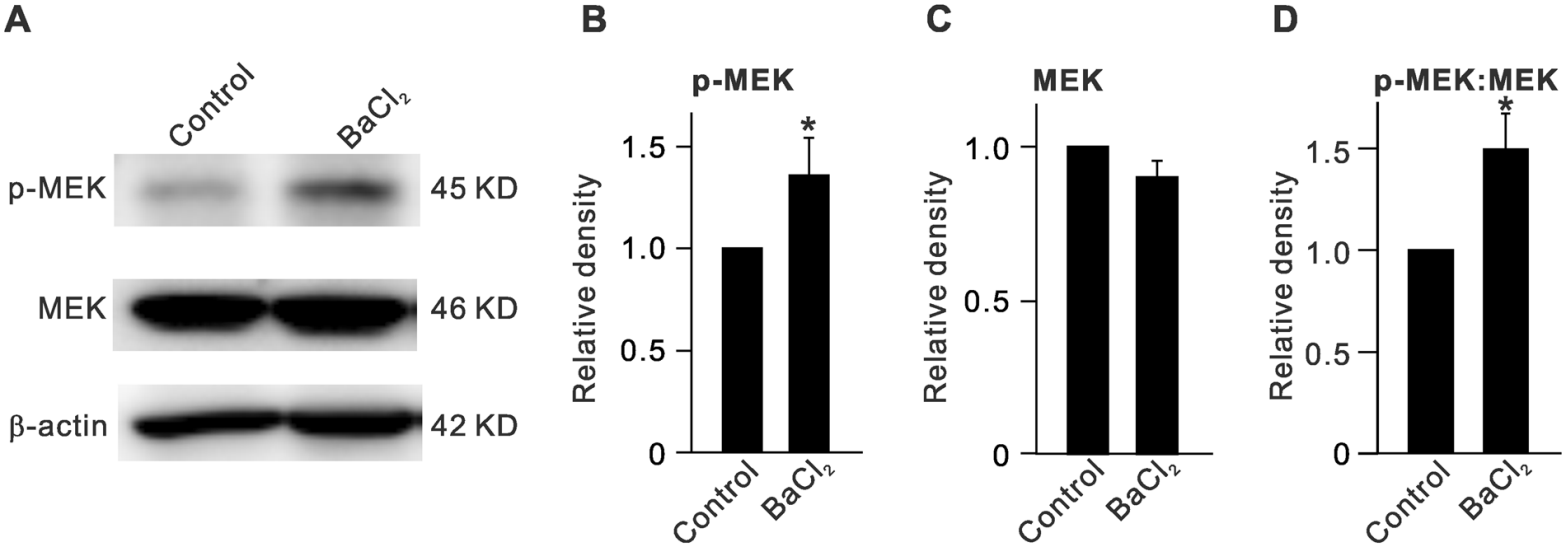
Elevation of p-MEK expression in BaCl_2_-injected retinas. (**A**) Representative immunoblots showing changes in p-MEK and MEK expressions in retinas with vehicle (Control)- or BaCl_2_-injection. (**B**,**C**) Bar charts summarizing the average densitometric quantification of immunoreactive bands of p-MEK (**B**) and MEK (**C**) protein expression as shown in (**A**). (**D**) Bar charts summarizing the average p-MEK:MEK ratio. *n* = 6 all groups. *P < 0.05 *vs*. Control.

Next, we examined changes in protein levels of MEK, an upstream regulator of ERK, after inhibiting Kir channels. Intravitreal injection of BaCl_2_ induced an increase in p-MEK protein levels (135.8 ± 18.5% of control (n = 6, *P* = 0.029) (Fig. 3A,B), while total MEK protein levels did not change significantly (90.0 ± 5.4% of control, n = 6, *P* = 0.384) (Fig. 3A,C), which resulted in an increased p-MEK:MEK ratio (151.4 ± 17.9% of control, n = 6, *P* = 0.043) (Fig. 3D).

### Inhibition of Kir channels increases p-CREB and c-fos levels in BaCl_2_-injected retinas

**Figure 4:**
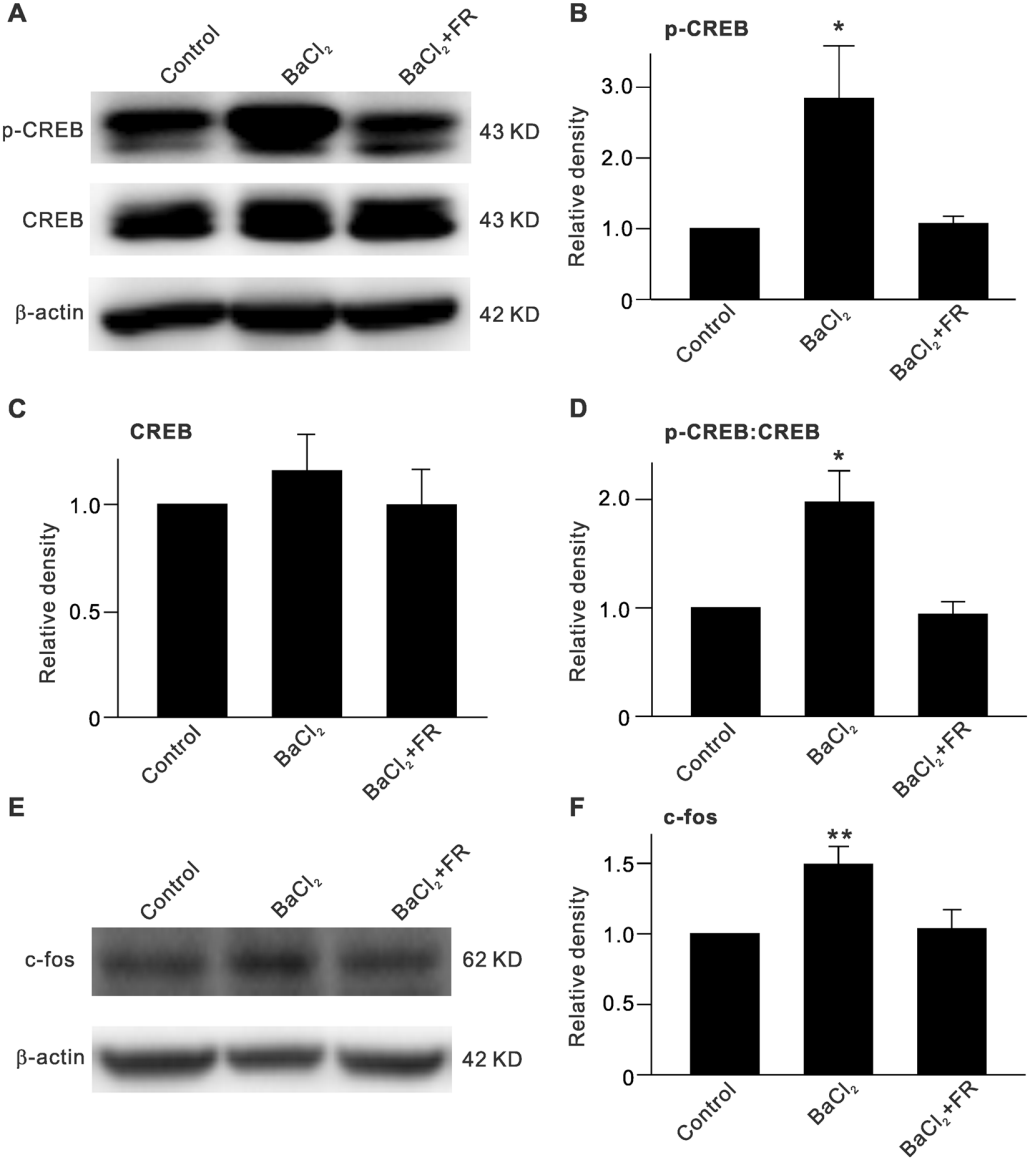
ERK-induced increase in p-CREB and c-fos expression in BaCl_2_-injected retinas. (**A**) Representative immunoblots showing changes in p-CREB and CREB expressions in retinas with vehicle (Control)-, BaCl_2_- or BaCl_2_ + FR180204-injection. (**B**,**C**) Bar charts summarizing the average densitometric quantification of immunoreactive bands of p-CREB (**B**) and CREB (**C**) protein expression under different conditions. (**D**) Bar charts summarizing the average p-CREB:CREB ratios under different conditions. (**E**) Representative immunoblots showing changes in c-fos expression. (**B**) Bar charts summarizing the average densitometric quantification of immunoreactive bands of c-fos protein expression under different conditions. Note that BaCl_2_ injection-induced increase in protein levels of p-CREB and c-fos, and ratio of p-CREB:CREB was reversed by co-injection of the ERK inhibitor FR180204. *n* = 6 for each group. *P < 0.05 and **P < 0.01 *vs*. Control.

p-ERK can translocalize into the nucleus and activate its downstream targets, including cAMP response element binding protein (CREB) and c-fos, thus influencing transcription, translation, and protein synthesis. We first examined changes in expression of p-CREB in BaCl_2_-injected retinas. As shown in Fig. 4, BaCl_2_ injection did not affect total CREB levels, but profoundly increased p-CREB levels to 284.2 ± 74.3% of control (n = 6, *P* = 0.023) as well as the p-CREB:CREB ratio to 197.3 ± 29.2% of control (n = 6, *P* = 0.048). In addition, FR180204, a selective ERK inhibitor, which was co-injected with BaCl_2_, rescued the enhanced p-CREB levels and p-CREB:CREB ratio to control levels (Fig. 4C,D). Similarly, c-fos protein levels were increased by BaCl_2_-injection (149.3 ± 12.6% of control, n = 6, *P* = 0.008) and were reversed by FR180204 (Fig. 4E,F).

### Inhibition of Kir channels enhances GFAP expression by activating MEK-ERK/p38-CREB/c-fos signaling in purified cultured Müller cells

**Figure 5:**
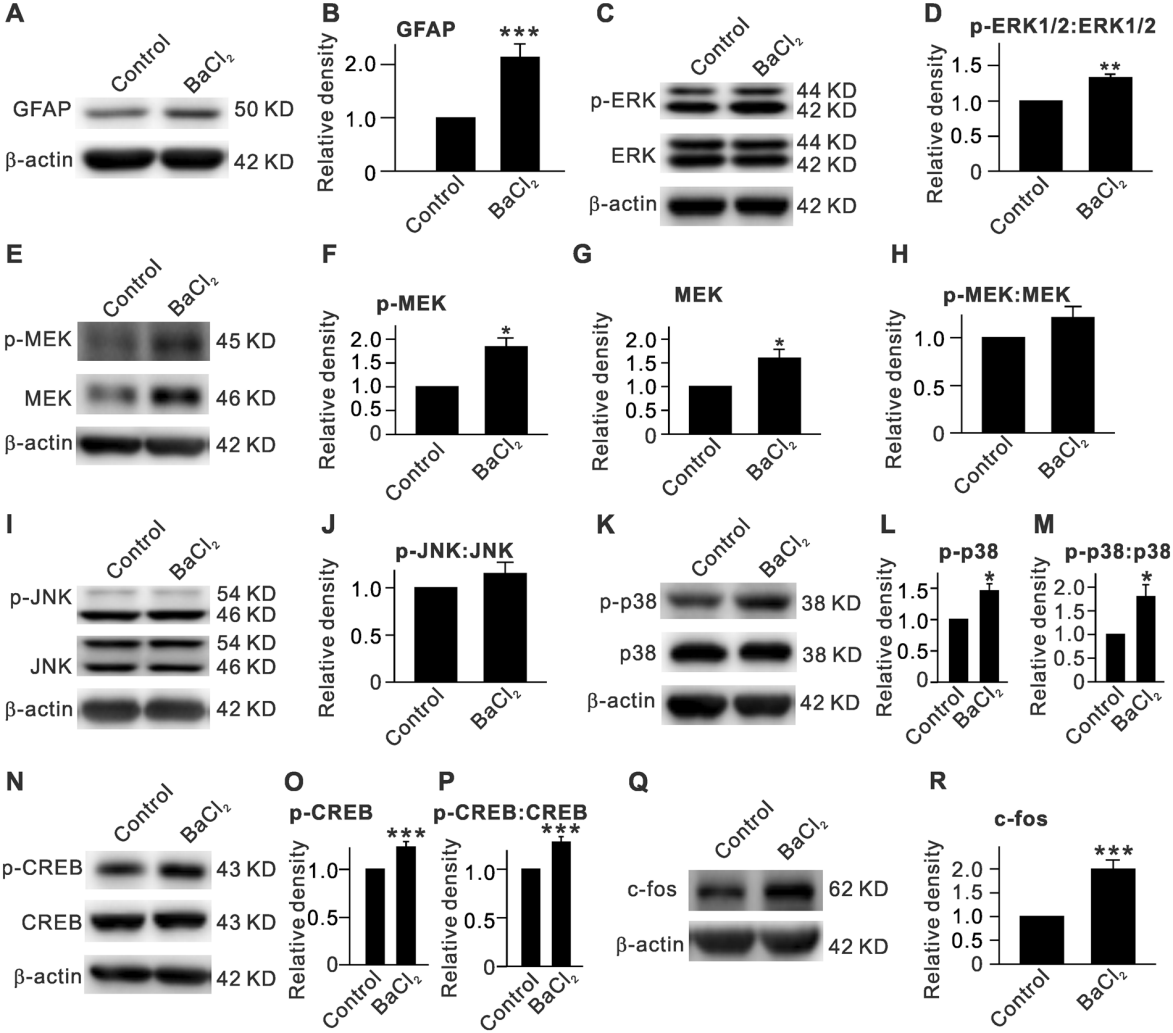
BaCl_2_ treatment induced upregulation of GFAP expression and changes in protein levels of p-ERK, p-MEK, p-JNK, p-p38, p-CREB and c-fos in purified cultured rat retinal Müller cells. (**A**) Representative immunoblots showing the changes in GFAP protein level assayed by Western blotting. Müller cells were treated with 20 µM BaCl_2_ for 24 h. (**B**) Bar chat summarizing the average densitometric quantification of immunoreactive bands of GFAP. (**C**) Representative immunoblots showing the changes in p-ERK1/2 and ERK1/2 protein levels. (**D**) Bar chat summarizing the average p-ERK1/2:ERK1/2 ratios. (**E**–**H**) Representative immunoblots showing the changes in p-MEK and MEK protein levels (**E**), and bar chat summarizing the average densitometric quantification of immunoreactive bands of p-MEK (**F**), MEK (**G**), and the average p-MEK:MEK ratio (**H**). (**I**,**J**) Representative immunoblots showing the changes in p-JNK and JNK protein levels (**I**), and bar chat summarizing the p-JNK:JNK ratio (**J**). (**K**–**M**) Representative immunoblots showing the changes in p-p38 and p38 protein levels (**K**), and bar chat summarizing the average densitometric quantification of immunoreactive bands of p-p38 (**L**) and the average p-p38:p38 ratio (**M**). (**N**–**P**) Representative immunoblots showing the changes in p-CREB and CREB protein levels (**N**), and bar chat summarizing the average densitometric quantification of immunoreactive bands of p-CREB (**O**) and the average p-CREB:CREB ratio (**P**). (**Q**,**R**) Representative immunoblots showing the changes in c-fos protein level (**Q**), and bar chat summarizing the average densitometric quantification of immunoreactive bands of c-fos (**R**). n = 9 for each group. *P < 0.05, **P < 0.01 and ***P < 0.001 *vs*. Control.

We further examined whether BaCl_2_ treatment could induce cell gliosis in purified cultured rat Müller cells, and the possible involvement of MEK-ERK-CERB/c-fos signaling was also investigated. Representative Western blot results showing the expression levels of GFAP after BaCl_2_ (20 µM) treatment for 24 h are shown in Fig. 5A. BaCl_2_ treatment significantly increased the GFAP levels to 213.8 ± 25.1% of control (n = 9, *P* < 0.001) (Fig. 5B). Similar to the observations in BaCl_2_-injected retinas, p-ERK1/2 (149.4 ± 5.8% of control, n = 9, *P* < 0.001), the p-ERK1/2:ERK1/2 ratio (133.0 ± 5.3% of control, n = 9, *P* = 0.008), and p-MEK levels (184.0 ± 18.4% of control, n = 9, *P* = 0.020) were considerably increased in BaCl_2_-treated cells (Fig. 5C–F), while total ERK1/2 protein levels remained unchanged (107.6 ± 3.0% of control, n = 9, *P* = 0.160). However, it is of interest to note that in BaCl_2_-treated Müller cells, MEK protein levels were increased (159.8 ± 18.8% of control, n = 9, *P* = 0.013), which resulted in a moderate elevation of the p-MEK:MEK ratio (120.9 ± 11.7% of control, n = 9, *P* = 0.335) that was not different than control levels (Fig. 5G,H).

Changes in protein levels of JNK, p-JNK, and of the p-JNK:JNK ratio (n = 9, *P* all >0.05) (Fig. 5I,J) in BaCl_2_-treated Müller cells were similar to those in BaCl_2_-injected retinas. In contrast, BaCl_2_-treatment induced a significant increase in p-p38 protein levels (145.9 ± 11.2% of control, n = 9, *P* = 0.028) and in the p-p38:p38 ratio (179.9 ± 25.4% of control, n = 9, *P* = 0.039) in cultured Müller cells (Fig. 5K–M). Furthermore, in BaCl_2_-treated Müller cells, changes in CREB, p-CREB, and c-fos protein levels as well as the p-CREB:CREB ratio were similar to those in BaCl_2_-injected retinas (Fig. 5N–R).

### Inhibition of the MAPK/ERK signaling pathway reduces GFAP expression in COH retinas

**Figure 6:**
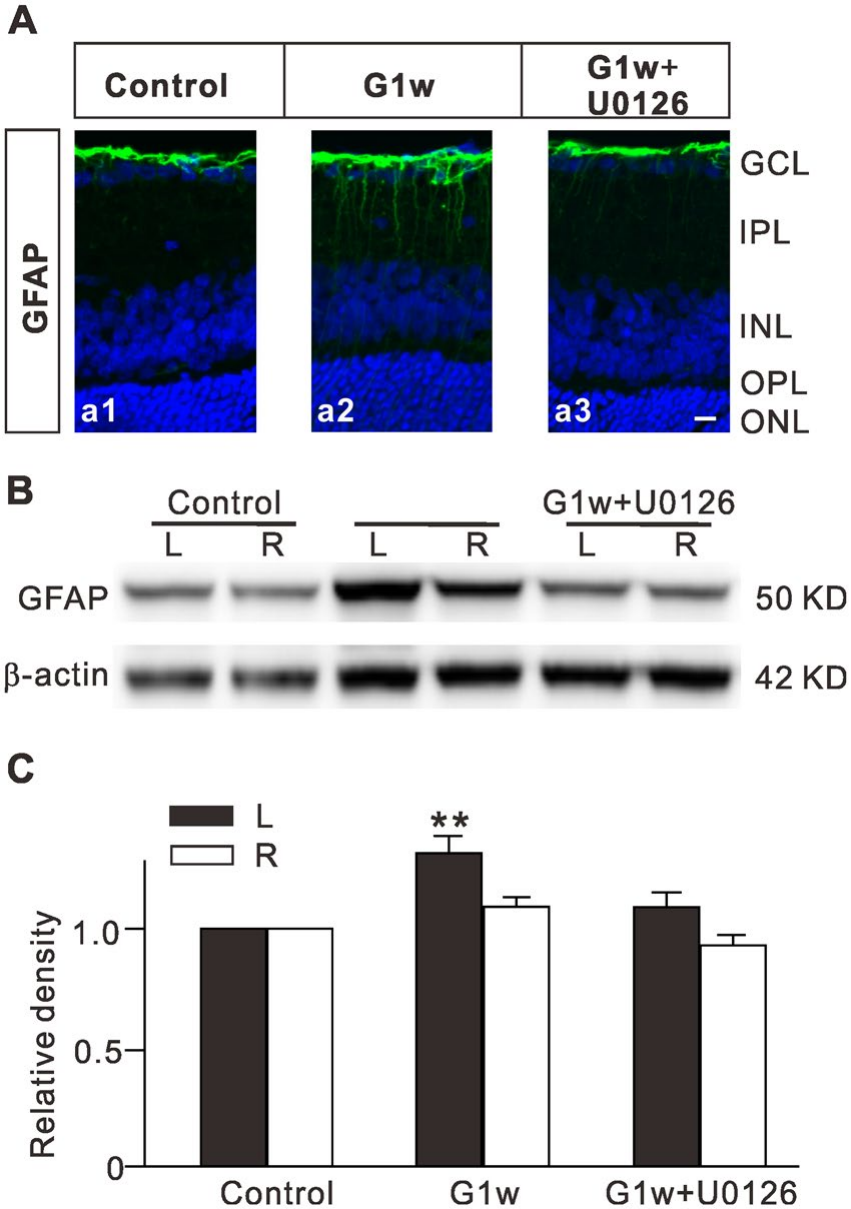
Intravitreal injection of U0126 inhibits upregulation of GFAP expression in Müller cells of retinas with COH. (**A**) Immunofluorescence labeling showing GFAP expression profiles (green) in rat retinal vertical sections, taken from sham-operated retina (control) (a1), COH retina 1 week after the operation (G1w) (a2), and COH retina with U0126 injection (a3). U0126 (10 µM, 2 µL) was intravitreally injected 3 days prior to the COH operation. (**B**) Representative immunoblots showing the changes in GFAP levels in control and COH retinal extracts obtained from both operated eyes and their unoperated contralateral eyes with or without U0126 injection. (**C**) Bar charts summarizing the average densitometric quantification of immunoreactive bands of GFAP protein expression under different conditions. *n* = 6 for each group. **P < 0.01 *vs*. left eyes in control.

Finally, we tested whether inhibition of MAPK/ERK signaling affected Müller cell gliosis in COH retinas. The rat COH model was used, as our previous reports^11, 32, 33^. The average IOPs of operated eyes from day 1 to 1 week (G1d to G1w) ranged from 24.8 ± 0.3 to 25.5 ± 1.6 mmHg (n = 12), which was significantly higher than that for unoperated eyes (19.0 ± 0.3 mmHg, n = 12) and for sham-operated eyes (19.0 ± 0.6 mmHg, n = 6) (*P* all <0.001). Fig. 6A shows that GFAP expression was significantly increased in retinal vertical section obtained from COH rat at G1w (a2), as compared to that of sham-operated rat (control) (a1), which is consistent with our previous study^11^. Intravitreal injection of U0126 3d prior to the COH operation eliminated the increase of GFAP expression in COH retina (a3). Consistently, the retinal GFAP level, assessed by Western blotting, was increased to 131.6 ± 7.4% of control (n = 6, *P* = 0.002) in operated left eyes (Fig. 6B,C). Intravitreal injection of U0126 completely eliminated the IOP elevation-induced upregulation of GFAP levels (109.0 ± 6.2% of control, n = 6, *P* = 0.146) (Fig. 6B,C). These results strongly suggest that the MAPK/ERK signaling pathway is involved in Müller cell gliosis.

## Discussion

Increasing evidence has demonstrated that downregulation of Kir channels is a key step for Müller cell gliosis in experimental glaucoma models and in patients with glaucoma^6, 11, 18, 22^. It is commonly believed that inhibition of Kir channels leads to Müller cell depolarization^6, 23, 27, 28^, which results in a loss of the strongly hyperpolarized resting potential that is important for the physiological functions of these cells. In this study, we found that intravitreal injection of BaCl_2_ induced Müller cell gliosis in rat retinas, similar to our previous report^11^. Since we have demonstrated that the BaCl_2_-induced GFAP upregulation was due to inhibition of Kir channels, and not by its nonspecific effects^11^, it is reasonable to say that intravitreal injection of BaCl_2_ resulting in Kir channel inhibition is an effective method for the induction of Müller cell gliosis. It should be noted that Ba^2+^ is not a selective Kir channel blocker. Previous study has shown that Ba^2+^ could also inhibit hyperpolarization-activated cation current (*I*_h_) channels in a low affinity manner in rat hippocampal CA1 pyramidal neurons^34^. Modulation of *I*_h_ channels may affect the neuronal excitability in retina^35^. However, there is no evidence showing that *I*_h_ channels are expressed in retinal Müller cells. Therefore, Ba^2+^-induced Müller cell gliosis was indeed by inhibiting Kir channels in Müller cells.

Our work provides direct evidence showing that the MEK-ERK-CREB/c-fos signaling pathway mediated Kir channel inhibition-induced upregulation of GFAP expression in rat Müller cells. This is supported by the following facts. First, the MEK inhibitor U0126 completely inhibited BaCl_2_ injection-induced GFAP upregulation in retinal Müller cells, as shown using immunohistochemistry and Western blotting (Fig. 1). Next, the protein levels of p-ERK1/2 and p-MEK, and the ratios of p-ERK1/2:ERK1/2 and p-MEK:MEK were significantly increased in BaCl_2_-injected retinas (Figs 2–4). U0126, but not U0124, reversed the effects of BaCl_2_ on protein levels of p-ERK1/2 and the p-ERK1/2:ERK1/2 ratio, suggesting that the activity of the MEK/ERK pathway was enhanced. In addition, it should be noted that total and phosphorylated protein levels of JNK and p38, another two types of MAPKs, did not show significant changes in BaCl_2_ injected retinas. Indeed, a previous study has shown that in retinas of patients with glaucoma, p-ERK, but not p-JNK and p-p38, was detected predominately in Müller cells although the intensity of immunostaining for the MAPKs and the number of MAPK-positive cells were greater than those of control eyes^30^. In a rat retinal ischemia-reperfusion model induced by acutely elevating IOP to 110 mmHg for 45–60 min, expression of p-ERK in Müller cells increased significantly^29^. Furthermore, intravitreal injection of BaCl_2_ induced the upregulation of p-CREB and c-fos protein levels as well as the p-CREB:CREB ratio. The BaCl_2_-induced effects on p-CREB and c-fos expression were reversed by FR180204, a specific ERK inhibitor, strongly suggesting that the elevation of p-CREB and c-fos expression was triggered by p-ERK. It has been shown that ERK plays an important role in regulating neuronal functions^36^. CREB can be phosphorylated not only by protein kinase A (PKA), but also by other kinases, such as ERK^37^. ERK can be activated by numerous stimuli (in this study it was stimulated by inhibition of Kir channels by BaCl_2_); in turn, p-ERK may activate its downstream targets including CREB and c-fos. Activating the p-ERK/p-CREB signaling pathway affects translation and new protein synthesis^38, 39^. Usually, the basal level of c-fos expression in neurons is low, but it can be elevated following stimulation through second messenger systems^40^. Activated c-fos can bind to DNA and regulate the transcription of various target genes, such as GFAP. Therefore, it is reasonable to deduce that p-ERK increased the activities of p-CREB and c-fos, thus increasing GFAP protein synthesis.

In addition to Müller cells, Kir channels are also expressed in retinal neurons, such as RGCs^35, 41^. To exclude the possible involvement of these retinal neurons in BaCl_2_-induced upregulation of GFAP expression, purified cultured rat Müller cells were treated with BaCl_2_ and changes in GFAP and MAPK protein levels were examined by Western blotting. Our results clearly showed that BaCl_2_ treatment of cultured Müller cells induced an increase in GFAP expression, suggestive of Müller cell gliosis. Significant upregulation of p-ERK1/2, p-MEK, p-CREB, and c-fos protein levels, as well as p-ERK1/2:ERK1/2 ratios, was detected in BaCl_2_-treated cells, confirming the involvement of the MEK-ERK-CREB/c-fos signaling pathway by Kir channel inhibition. However, it should be noted that in BaCl_2_-treated Müller cells, the p-p38 protein levels and the p-p38:p38 ratio were also significantly increased, which was inconsistent with the observations from BaCl_2_-injected retinas. Two factors may contribute to this inconsistency. First, among the three major types of MAPKs, ERK is expressed mainly in glial cells, while p38 and JNK are localized in RGCs and amacrine cells^29, 30^. Under pathological conditions, elevated levels of p-ERK were detected in Müller cells, and p-JNK and p-p38 were associated with nonglial cells^29, 30^. In addition, some faint p-p38 was also detectable in scattered glial cells or their processes^29^. In BaCl_2_-injected retinas, changes in p-p38 may occur only in Müller cells. Therefore, in whole retinal homogenates obtained from BaCl_2_-injected rats, p-p38 protein levels may be too low to be detected. Second, in BaCl_2_-injected retinas, Ba^2+^ may inhibit Kir channels in retinal neurons, and subsequently influence p-p38 protein levels in these neurons, resulting in unchanged total p-p38 levels and p-p38:p38 ratio in whole retinal extracts. The elevated level of p-p38 appeared when retinal neurons were absent in purified Müller cell cultures. Nevertheless, upregulation of GFAP expression in Müller cells induced by Kir channel inhibition may be mediated by increased p-ERK and p-p38, with p-ERK being the predominant mediator. Furthermore, in BaCl_2_-treated Müller cells, total MEK protein levels were increased in addition to upregulated p-MEK. This resulted in an unchanged p-MEK:MEK ratio, even though p-MEK was significantly increased. We speculate that this may be due to the continuous strong depolarization induced by BaCl_2_ treatment. This issue should be investigated in our future studies. In addition, a remained question is how to link Müller cell depolarization to MEK/ERK signaling activation. It was reported that MEK/ERK signaling pathway could be activated by elevating intracellular Ca^2+^ in neurons^42^. Voltage-gated Ca^2+^ channels, such as T- and L-type Ca^2+^ channels, were functionally expressed in retinal Müller cells^43^. Inhibition of Kir channels by Ba^2+^ could result in membrane depolarization of Müller cells, in turn activate voltage-gated Ca^2+^ channels and increase Ca^2+^ influx, thus activating Ca^2+^-dependent MEK/ERK signaling.

It is noteworthy that in the retina, different types of MAPKs may have distinct functions depending on their differential expression in different cells. The ERK signaling pathway is generally activated by extracellular stimuli and other factors, such as mitogens. ERK signaling is involved in modulation of transcriptional activity, thus influencing cell growth and differentiation^44^. In this work, we found that inhibition of Kir channels activates ERK signaling, as evidenced by increased p-ERK levels. p-ERK further activates CREB and c-fos, thus increasing GFAP protein synthesis. On the other hand, the p38 and JNK pathways are strongly activated by cytokines, such as tumor necrosis factor (TNF)-α^45^, which have been implicated in neuronal death^46–50^. For instance, p38 was involved in axotomized RGC death in chick embryos^47^, and in RGC apoptosis mediated by glutamate neurotoxicity in rats^50, 51^. Blocking the apoptosis signal-regulating kinase 1 (ASK1)-p38 pathway could prevent RGC death following optic nerve injury^52^. In addition, previous studies have demonstrated that JNK may play a major role in various forms of neuronal death. In the rat retinal ischemia-reperfusion model, inhibition of JNK activation significantly decreased cell apoptosis in the GCL, the inner nuclear layer (INL), and the photoreceptor layer^53^. In mice, JNK signaling was activated after axonal injury of RGCs, which may be the major early pathway triggering RGC death after axonal injury, and may directly link axonal injury to the transcriptional activity that controls RGC death^54^. In BaCl_2_-injected retinas, p38 and JNK signaling pathways remained unchanged, suggesting that inhibition of Kir channels in retinal neurons was not sufficient to activate these two pathways, even though it was sufficient to strongly activate ERK in Müller cells. Moreover, inhibition of ERK signaling pathway by intravitreal injection of U0126 eliminated GFAP expression upregulation in COH retinas, further demonstrating the involvement of ERK signaling pathway in Müller cell gliosis.

In conclusion, we here demonstrate that the MEK/ERK-p38/CREB-c-fos signaling pathway mediates the Kir channel inhibition-induced upregulation of GFAP in rat Müller cells. Previous studies have demonstrated that activated Müller cells may release cytotoxic factors, such as TNF-α and nitric oxide (NO), which acted on RGCs and resulted in RGC damage^6^. Our present results suggest that appropriate reduction of MEK/ERK signaling pathway could alleviate Müller cell gliosis, which may be an effective way for preventing the loss of RGCs in glaucoma.

## Methods

### Animals

All experimental procedures described were approved by the Laboratory Animal Care and Use Committee at Fudan University (Shanghai, China) and are in accordance with the National Institutes of Health (NIH) guidelines. Male Sprague-Dawley rats, purchased from SLAC Laboratory Animal Co., Ltd (Shanghai, China), were maintained on a 12-h light/dark cycle.

### Intravitreal injection

The intravitreal injection procedure was performed as previously described^11, 32^. Briefly, the pupil of the anesthetized eye was dilated with tropicamide drops. BaCl_2_ (200 µM), U0126 (10 µM), U0124 (10 µM), or FR180204 (1 µM) dispersed in 2 μL of 0.9% saline were injected into the vitreous space through a post limbus spot using a Hamilton microinjector (Hamilton, Reno, NV, USA) under a stereoscopic microscope (Carl Zeiss, Jena, Germany).

### Purified retinal Müller cell culture

Retinal Müller cell cultures were prepared following the procedure previously described in detail^22, 55^, with minor modifications. The retinas of newborn Sprague-Dawley rats (5-d old) were digested with 0.25% trypsin in a Ca^2+^ and Mg^2+^-free D-Hank’s solution supplemented with HEPES (10 mM) for 15 min at 37 °C. The cell suspension was plated onto poly-D-lysine pre-coated 25 cm^2^ flasks at a density of 1 × 10^6^, and cultured in Dulbecco’s modified eagle medium (DMEM/F12; Gibco, Life Technologies, Rockville, MD, USA) supplemented with 10% fetal bovine serum (FBS) in a humidified 5% CO_2_ incubator at 37 °C. Non-attached cells and microglia cells were removed by gently blowing with a fire-polished Pasteur pipette when the culture medium was changed every 3 d. The third-generation of retinal Müller cells, cultured for up to 21 d, was used for experiments.

### Rat COH model

COH rats were produced following the procedure previously described in detail^11, 32, 33^. Briefly, in anesthetized rats, three episcleral veins of the left eye were ligated and cauterized. Intraocular pressure (IOP) was measured using a handheld digital tonometer (Tonolab, TioLat, Finland) under general and local anesthesia as described above. The average value of five consecutive measurements with a deviation of less than 5% was accepted. All measurements were performed in the morning to avoid possible circadian differences. The IOPs of both eyes were measured before surgery as a baseline, immediately after surgery (day 0), and on the third and seven days after surgery (day 3 and 1w, respectively).

### Immunohistochemistry

Immunohistochemistry was performed as described in our previous studies^11, 32, 33^, with minor modifications. Retinal vertical sections were cut at a thickness of 14 μm on a freezing microtome (Leica, Nussloch, Germany). The sections were blocked in 5% normal donkey serum and 1% bovine serum, dissolved in PBS plus 0.4% Triton X-100 at room temperature for 2 h, and then incubated with monoclonal mouse anti-GFAP primary antibody (1:400 dilution, Sigma-Aldrich, St. Louis, MO, USA) overnight at 4 °C. The binding sites of the primary antibody were visualized by incubating with 488-conjugated donkey anti-mouse IgG (1:400 dilution, Sigma-Aldrich) for 2 h at room temperature. The sections were visualized and photographed with a Leica SP2 confocal laser-scanning microscope.

### Western blot analysis

Western blot analysis was conducted as previously described with some modifications^11, 32, 33, 56^. Briefly, the extracted protein samples (1.0 μg/μL, 10 or 15 μL) were resolved using 10% SDS-PAGE gels and transferred onto polyvinylidene fluoride (PVDF) membranes (Immobilon-P, Millipore, Billerica, MA, USA) using the Mini-PROTEAN 3 Electrophoresis System and the Mini Trans-Blot Electrophoretic Transfer System (Bio-Rad, Hercules, CA, USA). The membranes were blocked in 5% non-fat milk (for non-phosphorylated antibodies) or in 5% bovine serum albumin (for phosphorylated antibodies) for 1 h at room temperature, and subsequently incubated with primary antibodies at 4 °C overnight. The following primary antibodies were used: monoclonal mouse anti-β-actin (1:3000 dilution, Sigma-Aldrich), monoclonal mouse anti-GFAP (1:1000 dilution, Sigma-Aldrich), monoclonal rabbit anti-GAPDH (1:1000 dilution, Cell Signaling Technology), monoclonal mouse anti-p-ERK1/2 (1:4000 dilution, Sigma-Aldrich), polyclonal rabbit anti-ERK (1:500 dilution, Santa Cruz Biotechnology), monoclonal mouse anti-p-p38 (1:200 dilution, Santa Cruz Biotechnology), polyclonal rabbit anti-p38α (1:500 dilution, Santa Cruz Biotechnology), monoclonal mouse anti-p-JNK (1:500 dilution, Santa Cruz Biotechnology), polyclonal rabbit anti-JNK (1:500 dilution, Santa Cruz Biotechnology), monoclonal rabbit anti-CREB (1:1000 dilution, Sigma-Aldrich), monoclonal mouse anti-p-CREB (1:1000 dilution, Cell Signaling Technology), monoclonal mouse anti-c-fos (1:200 dilution, Abcam), polyclonal rabbit anti-MEK (1:20,000 dilution, Sigma-Aldrich), and polyclonal rabbit anti-p-MEK (1:1000 dilution, Sigma-Aldrich). After washing in Tris-buffered saline-Tween 20, the membranes were incubated with horseradish-peroxidase (HRP)-conjugated goat anti-mouse or goat anti-rabbit IgG (1:5000; Jackson, Immunoresearch Laboratories, Wes Grove, PA, USA) for 1 h at room temperature. The blots were then incubated with enhanced chemiluminescent reagent (Thermo Scientific, Rockford, IL, USA) and imaged with a digital imager (FluorChem E System, ProteinSimple, USA). For sequential immunoblotting, the blots were washed with Tris-buffered saline, stripped with Restore Western Blot Stripping Buffer (Thermo Scientific, Rockford, IL, USA), and re-blocked and incubated with primary antibodies if necessary.

### Data analysis

Data were analyzed using GraphPad Prism software (version 5.0; GraphPad Software, San Diego, CA, USA) and values are expressed as mean ± SEM. A one-way analysis of variance (ANOVA) with Bonferroni’s post-hoc test (multiple comparisons) or the *t* test (unpaired data) was used. A value of *P* < 0.05 was considered to indicate statistical significance.

## References

[1] Guo L (2005). Retinal ganglion cell apoptosis in glaucoma is related to intraocular pressure and IOP-induced effects on extracellular matrix. Invest. Ophthalmol. Vis. Sci..

[2] Hitchings RA (2000). Selective ganglion cell death in glaucoma. Br. J. Ophthalmol..

[3] Resnikoff S (2004). Global data on visual impairment in the year 2002. Bull. World Health Organ..

[4] Gupta N, Yucel YH (2007). Glaucoma as a neurodegenerative disease. Curr. Opin. Ophthalmol..

[5] Bringmann A (2009). Cellular signaling and factors involved in Müller cell gliosis: neuroprotective and detrimental effects. Prog. Retin. Eye Res..

[6] Bringmann A (2006). Müller cells in the health and diseased retina. Prog. Retin. Eye Res..

[7] Goureau O, Regnier-Ricard F, Courtois Y (1999). Requirement for nitric oxide in retinal neuronal cell death induced by activated Müller glial cells. J. Neurochem..

[8] Kashiwagi K, Lizuka Y, Araie M, Suzuki K, Tsukahara S (2001). Effects of retinal glial cells on isolated rat retinal ganglion cells. Invest. Ophthalmol. Vis. Sci..

[9] Tezel G, Li LY, Patil RV, Wax MB (2001). Tumor neurosis factor-alpha and its receptor-1 in the retina of normal and glaucomatous eyes. Invest. Ophthalmol. Vis. Sci..

[10] Tezel G, Wax MB (2003). Glial modulation of retinal ganglion cell death in glaucoma. J. Glaucoma.

[11] Ji M (2012). Group I mGluR-mediated inhibition of Kir channels contributes to retinal muller cell gliosis in a rat chronic ocular hypertension model. J. Neurosci..

[12] Wang X, Tay SS, Ng YK (2000). An immunohistochemical study of neuronal and glial cell reactions in retinae of rats with experimental glaucoma. Exp. Brain Res..

[13] Woldemussie E, Wijono M, Ruiz G (2004). Müller cell response to laser-induced increase in intraocular pressure in rats. Glia.

[14] Xue LP (2006). Müller glial cells express nestin coupled with glial fibrillary acidic protein in experimentally induced glaucoma in the rat retina. Neuroscience.

[15] Inman DM, Horner PJ (2007). Reactive nonproliferative gliosis predominates in a chronic mouse model of glaucoma. Glia.

[16] Bolz S (2008). K^+^ currents fail to change in reactive retinal glial cells in a mouse model of glaucoma. Graefes Arch. Clin. Exp. Ophthalmol..

[17] Zhang S (2009). Detection of early neuron degeneration and accompanying glial responses in the visual pathway in a rat model of acute intraocular hypertension. Brain Res..

[18] Frankcke M (1997). Loss of inwardly rectifying potassium currents by human retinal glial cells in disease of the eye. Glia.

[19] Bringmann A (2000). Role of glial K^+^ channels in outogeny and gliosis: a hypothesis based upon studies on Müller cells. Glia.

[20] Frankcke M (2000). Electrophysiology of rabbit Müller (glial) cells in experimental retinal detachment and PVR. Invest. Ophthalmol. Vis. Sci..

[21] Pannicke T (2006). Diabetes alters osmotic swelling and membrane characteristics of glial cells in rat retina. Diabetes.

[22] Gao F (2015). Group I metabotropic glutamate receptor agonist DHPG modulates Kir4.1 protein and mRNA in cultured rat retinal Müller cells. Neurosci. Lett..

[23] Kofuji P (2002). Kir potassium channel subunit expression in retinal glial cells: implications for spatial potassium buffering. Glia.

[24] Ishii M (1997). Expression and clustered distribution of an inwardly rectifying potassium channel, KAB-2/Kir4.1, on mammalian retinal Müller cell membrane: their regulation by insulin and laminin signals. J. Neurosci..

[25] Iandiev I (2006). Differential regulation of Kir4.1 and Kir2.1 expression in the ischemic rat retina. Neurosci. Lett..

[26] Wurm A (2006). The developmental expression of K^+^ channels in retinal glial cells is associated with a decrease of osmotic cell swelling. Glia.

[27] Pannicke T, Faude F, Reichenbach A, Reichelt W (2000). A function of delayed rectifier potassium channels in glial cells: maintenance of an auxiliary membrane potential under pathological conditions. Brain Res..

[28] Bringmann A (2002). Membrane conductance of Müller glial cells in proliferative diabetic retinopathy. Can. J. Ophthalmol..

[29] Roth S (2003). Mitogen-Activated Protein Kinases and Retinal Ischemia. Invest. Ophthalmol. Vis. Sci..

[30] Tezel G, Chauhan BC, LeBlanc RP, Wax MB (2003). Immunohistochemical assessment of the glial mitogen-activated. Invest. Ophthalmol. Vis. Sci..

[31] Dérijard B (1995). Independent human MAP kinase signal transduction pathways defined by MEK and MKK isoforms. Science.

[32] Dong LD (2015). GluA2 trafficking is involved in apoptosis of retinal ganglion cells induced by activation of EphB/ephrinB reverse signaling in a rat chronic ocular hypertension model. J. Neurosci..

[33] Chen J, Miao Y, Wang XH, Wang Z (2011). Elevation of p-NR2A^S1232^ by Cdk5/p35 contributes to retinal ganglion cell apoptosis in a rat experimental glaucoma model. Neurobiol. Dis..

[34] van Welie I, Wadman WJ, van Hooft JA (2005). Low affinity block of native and cloned hyperpolarization-activated Ih channels by Ba2+ ions. Eur. J. Pharmacol..

[35] Li Q (2017). Activation of group I metabotropic glutamate receptors regulates the excitability of rat retinal ganglion cells by suppressing Kir and Ih. Brain Struct. Funct..

[36] Shiflett MW, Balleine BW (2011). Contributions of ERK signaling in the striatum to instrumental learning and performance. Behav. Brain Res..

[37] Sun A (2015). Decrease of phosphorylated CREB and ERK in nucleus accumbens is associated with the incubation of heroin seeking induced by cues after withdrawal. Neurosci. Lett..

[38] Shaul YD, Seger R (2007). The MEK/ERK cascade: from signaling specificity to diverse functions. Biochim. Biophys. Acta.

[39] Xu Y (2012). Essential role of NR2B-containing NMDA receptor-ERK pathway in nucleus accumbens shell in morphine-associated contextual memory. Brain Res. Bull..

[40] Nathaniel TI, Panksepp J, Huber R (2012). Alteration of c-Fos mRNA in the accessory lobe of crayfish is associated with a conditioned-cocaine induced reward. Neurosci. Res..

[41] Chen L, Yu YC, Zhao JW, Yang XL (2004). Inwardly rectifying potassium channels in rat retinal ganglion cells. Eur. J. Neurosci..

[42] Dreses-Werringloer U (2013). CALHM1 controls the Ca^2+^-dependent MEK, ERK, RSK and MSK signaling cascade in neurons. J. Cell Sci..

[43] Yang W (2016). Cannabinoid receptor agonists modulate calcium channels in rat retinal muller cells. Neuroscience.

[44] Boulton TG (1991). ERKs: a family of protein-serine/threonine kinases that are activated and tyrosine phosphorylated in response to insulin and NGF. Cell.

[45] Mielke K, Herdegen T (2000). JNK and p38 stresskinases: degenerative effectors of signal-transduction-cascades in the nervous system. Prog. Neurobiol..

[46] Raingeaud J (1995). Pro-inflammatory cytokines and environmental stress cause p38 mitogen-activated protein kinase activation by dual phosphorylation on tyrosine and threonine. J. Biol. Chem..

[47] Kummer JL, Rao PK, Heidenreich KA (1997). Apoptosis induced by withdrawal of trophic factors is mediated by p38 mitogen-activated protein kinase. J. Biol. Chem..

[48] Xia Z, Dickens M, Raingeaud J, Davis RJ, Greenberg ME (1995). Opposing effects of ERK and JNK-p38 MAP kinases on apoptosis. Science.

[49] Herdegen T (1998). Lasting N-terminal phosphorylation of c-Jun and activation of c-Jun N-terminal kinases after neuronal injury. J. Neurosci..

[50] Castagne V, Clarke PG (1999). Inhibitors of mitogen-activated protein kinases protect axotomized developing neurons. Brain Res..

[51] Kikuchi M, Tenneti L, Lipton SA (2000). Role of p38 mitogen-activated protein kinase in axotomy-induced apoptosis of rat retinal ganglion cells. J. Neurosci..

[52] Katome T (2013). Inhibition of ASK1-p38 pathway prevents neural cell death following optic nerve injury. Cell Death Differ..

[53] Produit-Zengaffinen N, Favez T, Pournaras CJ, Schorderet DF (2016). JNK Inhibition Reduced Retinal Ganglion Cell Death after Ischemia/Reperfusion *In Vivo* and after Hypoxia *In Vitro*. Adv. Exp. Med. Biol..

[54] Fernandes KA (2012). JNK2 and JNK3 are major regulators of axonal injury-induced retinal ganglion cell death. Neurobiol. Dis..

[55] Hauck SM, Suppmann S, Ueffing M (2003). Proteomic profiling of primary retinal Müller glia cells reveals a shift in expression patterns upon adaptation to *in vitro* conditions. Glia.

[56] Miao Y (2012). Involvement of calpain/p35-p25/Cdk5/NMDAR signaling pathway in glutamate-induced neurotoxicity in cultured rat retinal neurons. PLoS One.

